# p53 mutation and deletion contribute to tumor immune evasion

**DOI:** 10.3389/fgene.2023.1088455

**Published:** 2023-02-20

**Authors:** Siyang Liu, Tianyao Liu, Jiaxuan Jiang, Hongqian Guo, Rong Yang

**Affiliations:** ^1^ Department of Urology, Affiliated Drum Tower Hospital, Medical School of Nanjing University, Nanjing, China; ^2^ Department of Endocrinology, Affiliated Drum Tower Hospital, Medical School of Nanjing University, Nanjing, China

**Keywords:** p53 mutation, p53 deletion, tumor immune evasion, tumor immune microenvironment, MHC

## Abstract

TP53 (or p53) is widely accepted to be a tumor suppressor. Upon various cellular stresses, p53 mediates cell cycle arrest and apoptosis to maintain genomic stability. p53 is also discovered to suppress tumor growth through regulating metabolism and ferroptosis. However, p53 is always lost or mutated in human and the loss or mutation of p53 is related to a high risk of tumors. Although the link between p53 and cancer has been well established, how the different p53 status of tumor cells help themselves evade immune response remains largely elusive. Understanding the molecular mechanisms of different status of p53 and tumor immune evasion can help optimize the currently used therapies. In this context, we discussed the how the antigen presentation and tumor antigen expression mode altered and described how the tumor cells shape a suppressive tumor immune microenvironment to facilitate its proliferation and metastasis.

## Introduction

**FIGURE 1:**
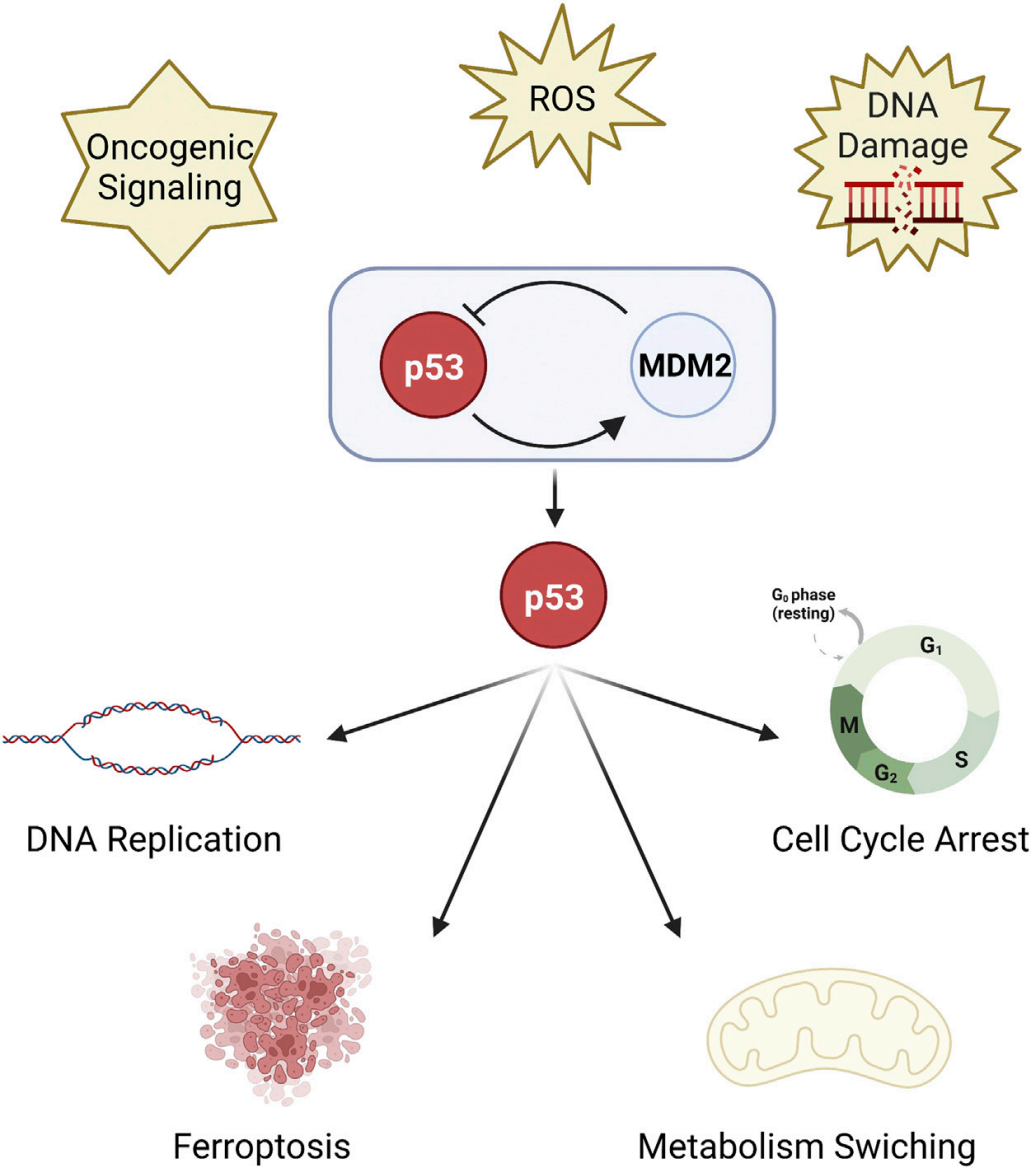
The function of p53. p53 is a key regulator of DNA replication stress and DNA repair. p53 is inhibited by MDM2 protein, but upregulated under stress. Upregulated p53 mediates cell cycle arrest and DNA replication, metabolism switching, and ferroptosis.

Genome instability is one of the hallmarks of cancer (Negrini et al., 2010; Hanahan and Weinberg, 2011). TP53 (or p53) is a vital tumor suppressor as it is the key regulator of DNA replication stress and DNA repair (Gaillard et al., 2015; Adriaens et al., 2016; Lindstrom et al., 2022) to maintain genomic stability. p53 responds to diverse cellular stresses, such as DNA damage, oxidative stress and oncogenic signaling (Hafner et al., 2019; Boutelle and Attardi, 2021). In unstressed, non-transformed cells, the expression and activity of p53 are blocked by its negative regulator MDM2 protein to be maintained at a low level (Haupt et al., 1997; Kubbutat et al., 1997; Shieh et al., 1997). On the contrary, the p53-MDM2 interaction will be lost and the expression of p53 is upregulated in stressed cells (Aubrey et al., 2018). Upregulated p53 mediates cell cycle arrest and apoptosis (Engeland, 2018) to eliminate damaged cells. p53 has a complicated link with the death or survival of tumor cells through regulating metabolism (Liu and Gu, 2021). Ferroptosis, an iron-dependent mode of death (Dixon et al., 2012) associated with metabolism, has also been recently found to be a p53-regulated activity to inhibit tumor growth (Jiang et al., 2015; Liu and Gu, 2022) (Figure 1).

The function of the immune system in control of cancer has been realized (Keast, 1970). Both the elements of innate immune and adaptive immune participate in anti-tumor activities, such as CD4^+^ T cells, CD8^+^ T cells, and natural killer (NK) cells. The immune response to cancer is thought to be activated in a tumor genome-dependent manner (Chen and Mellman, 2017). Tumor antigens originated from specific gene mutations (Epping and Bernards, 2006) are presented by dendritic (DC) cells or directly presented by tumor cells (Jhunjhunwala et al., 2021) for priming of CD8^+^ T cells to eliminate tumor cells. Simultaneously, tumor cells escape from immune attack through altering internal genes and shaping external environment, and p53 is one of the key points.

TP53 mutation is strongly associated with a risk of cancer (Harris, 1995; Willenbrink et al., 2020). Previous researches in transcriptome and proteome have demonstrated that TP53 mutation exists broadly in patients suffering tumors, such as urothelial carcinoma of the bladder (Xu et al., 2022), lung cancer (Chen and Roumeliotis, 2020; Gillette et al., 2020), and mutant p53 (hereafter referred to as “mutp53”) always results in poor prognosis (Cao et al., 2021). The mutp53 displays various responses in cellular activity (Muller and Vousden, 2013), mainly dominant-negative effects compared to wild-type p53 (wt-p53) (Muller and Vousden, 2014). The loss of p53 gene also results in developing more advanced carcinomas than p53^+/+^ and p53^+/−^ in mice skin cancer models (Guinea-Viniegra et al., 2012). Restoring the function or expression of p53 has been proved to inhibit tumor progression and even reduce tumor size in both *in vivo* and *in vitro* experiments (Ventura et al., 2007; Guinea-Viniegra et al., 2012).

Based on current research, the hallmarks of tumors with different status of p53 is clear, but how the tumor cells with different p53 status survived from immune surveillance remains largely elusive. Here, we focus on the complicated molecular network of tumor evasion derived from different status of p53 and explore new options of immunotherapy.

## The p53 mutation regulates the MHC molecules and reduces immunogenicity of tumor cells

Major histocompatibility complex (MHC) molecules expressed on cell surface present peptides to T cells to motivate immune responses. MHC molecules can be divided into two major classes. MHC Class I mainly presents peptides came from intracellular proteins (Jhunjhunwala et al., 2021), which prevent cells from malignant proliferation and stop cancer formation, while MHC Class II presents extracellular proteins to protect cells from infection. MHC I is formed by four domains. The α1, α2, and α3 domain form a heavy chain, and the β2m domain forms a light chain. After the heavy chain combine with β2m, the complex binds to peptides provided by the transporter associated with antigen processing (TAP) and is transported to the cell surface *via* the Golgi network (Flutter and Gao, 2004; Cresswell et al., 2005). Under most circumstances, tumor cells lack of the expression of MHC molecules to decrease their immunogenicity.

**FIGURE 2:**
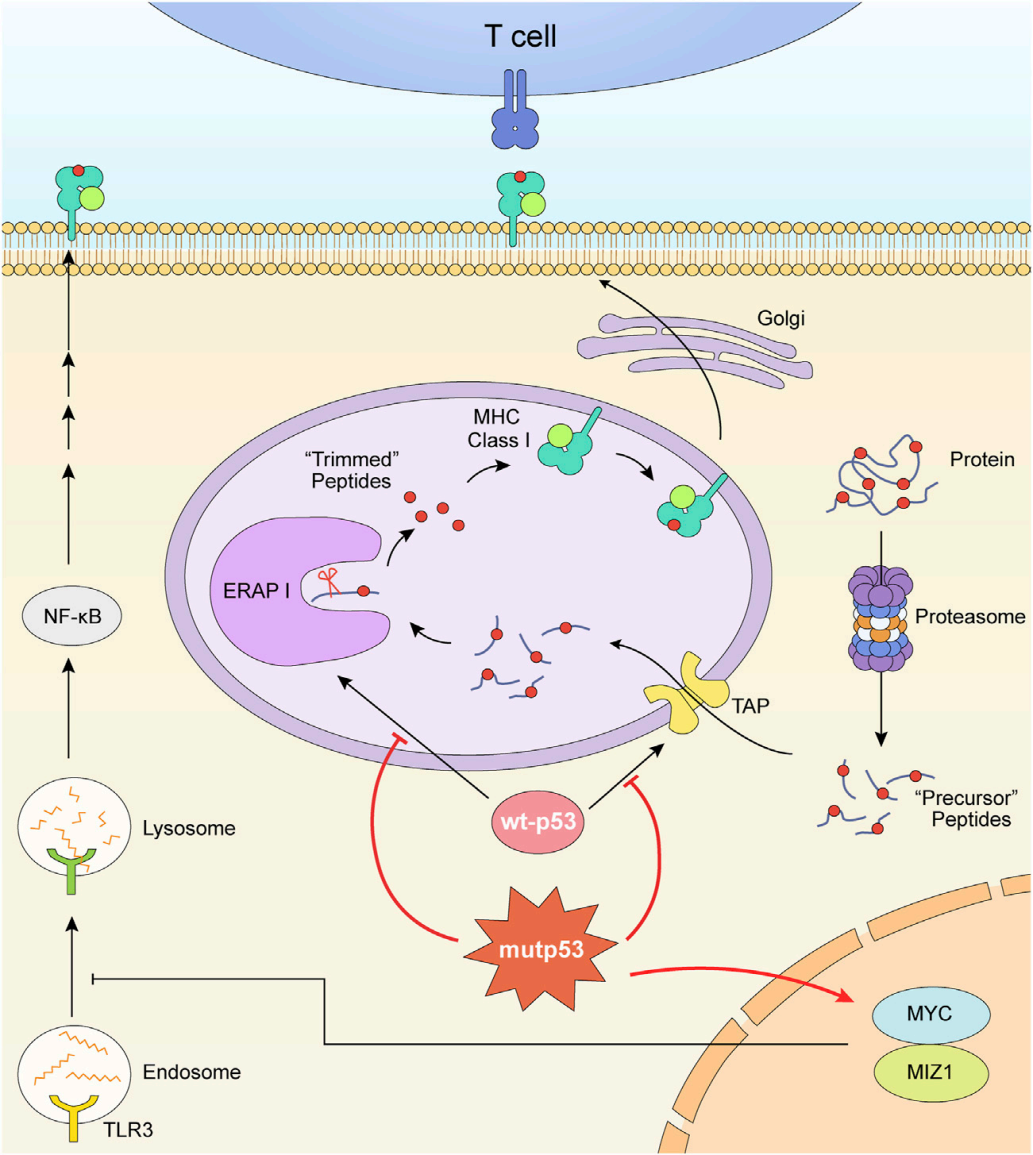
Mutant p53 downregulates the expression of MHC I. The formation and expression of MHC I is related with TAP and ERAP1. TAP transports peptides degraded by proteasome into ER. ERAP1 trims these precursor peptides to be optimal length for assembling with MHC. The dysfunctional p53 lost its TAP1 and ERAP1 activation function. What’s more, the p53 mutation and deletion play roles in upregulating MYC oncoprotein, consequently inhibited the activation of downstream MHC I.

Tumor cells that lack p53 exhibit markedly lower MHC I molecules (Bubeník, 2004) (Figure 2). The dysfunctional p53 lost its TAP1 activation function. In normal cells, TAP1 is induced by endogenous wild-type p53 (wt-p53) to enhance the transport and the expression of surface MHC-peptide complexes, but not in mutant p53 (R249S) cells and p53-null like HCT116E6 cells (Zhu et al., 1999). Similarly, endoplasmic reticulum aminopeptidase 1 (ERAP1) is another p53-target gene. ERAP1 acts as a molecular scissor to trim N-terminal extended peptides to be optimal length for assembling with MHC I (Falk and Rötzschke, 2002; Reeves et al., 2020). In human colon carcinoma cell lines, it has been proved that the cognate response element of ERAP1 gene is not accessible to bind silenced p53. The expression of MHC I consequently decreased. (Wang et al., 2013). Wt-p53 limits tumor growth *via* repressing the myelocytomatosis (Myc) oncogene transcription (Olivero et al., 2020). But the p53 deletion could increase the expression of Myc (Olivero et al., 2020) and the p53-R249S mutation could enhance the activity of Myc (Liao et al., 2017). Upregulated MYC prevented nuclear-derived double-stranded RNA from being recognized by toll-like receptor 3 (TLR3), consequently inhibited the activation of downstream MHC I (Krenz et al., 2021).

Thus, one of the treatments is to upregulate MHC I expression relying on the re-activation of p53. Pharmacologically activated p53 induced by MDM2 inhibitors enhanced the expression of endogenous retroviruses (ERV). The derepression of ERV triggered ERV-dsRNA-interferon (IFN) pathway followed by activation of antigen processing and presenting genes, including B2M, HLA-A, HLA-B, and HLA-C which encode MHC class I molecules (Zhou et al., 2021). Besides, it is also demonstrated that the dual-targeting PI3K and HDAC inhibitor BEBT-908 can promote ferroptosis of cancer cells by hyperacetylating p53 and promoting the expression of ferroptotic signaling (Liu et al., 2019; Fan et al., 2021). Acetylation-modified p53 in tumor cells induced the upregulation of MHC I *via* signal transducer and activator of transcription (STAT) one signaling pathway (Fan et al., 2021).

Another mechanism that p53 impacts immune escape is through affecting the recognition of MHC molecules. The different mutant p53 status interfere with TCR-MHC identification as endogenous proteins presented by MHC I *via* proteasome. By detecting the secretion of IFNγ, tumor necrosis factor (TNF)-α, and the proportion of CD69^+^ T cells, it was found that p53-bearing destabilizing mutations, such as R175H and Y220C mutations, are recognized more efficiently and activated more p53 target T cells than G245S mutation (Shamalov et al., 2017).

As for MHC II, it is found MHC II mediated the T-cell response to acute myeloid leukemia (AML) after allogeneic hematopoietic cell transplantation. The p53 deletion caused the downregulation of MHC II and tumor necrosis factor related apoptosis-inducing ligand receptor one and receptor 2 (TRAIL-R1/2) which induced immune evasion of AML (Chitlur, 2022; Ho et al., 2022).

## How does p53 mutation or deletion shape an immunosuppressive environment?

**FIGURE 3:**
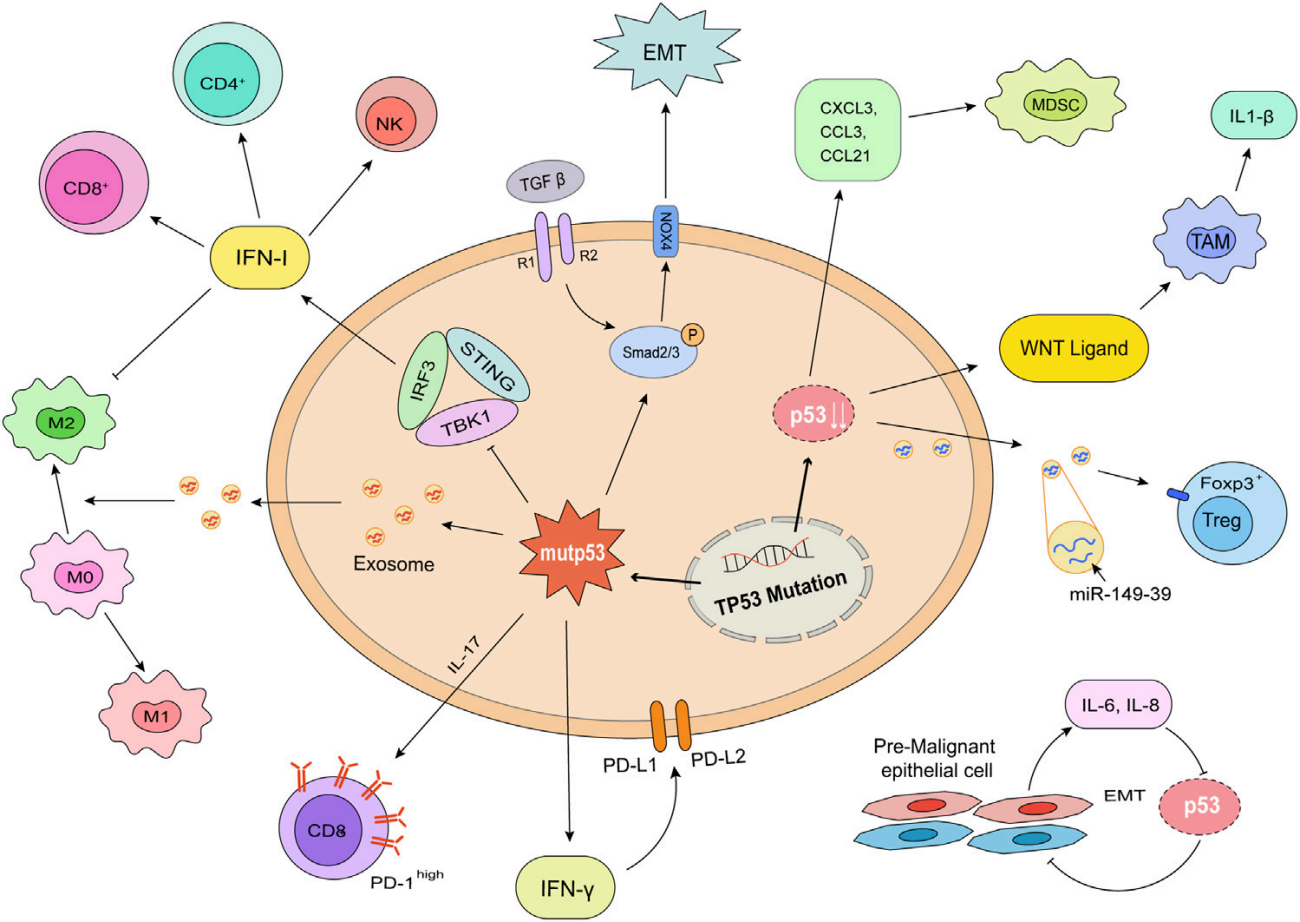
p53 mutation and deletion shape an immunosuppressive environment. The consequences of mutation (left) or deletion (right) of p53 in tumor cells are shown. Mutp53 decreased the release of IFN I, consequently decreased the infiltration of CD4^+^ T cells, CD8^+^ T cells, and NK cells. Mutp53 promoted the formation of exhausted CD8^+^ T cells through IL-17 signaling. The increased IFNγ played a role in the expression of immunosuppressive ligands, PD-L1 and PD-L2. Mutant p53 has a synergistic effect with TGF-β to promote EMT. p53 deletion upregulated the releasing of WNT ligands, CXCL1, CCL3, CCL21, and miR-149–39 to enhance the differentiation of TAMs and Tregs. Pre-malignant epithelial cells would release IL-6 and IL-8 to promote EMT. This process promoted tumor formation and metastasis.

Over the past few centuries, researchers came to realize tumors are more than a group of malignant proliferating cells, but a complex “organ” including tumor cells, stromal cells, immune cells, extracellular matrix, vessels, cytokines, chemokines, as well as other metabolic products (Fu et al., 2021). It has been found that tumor cells transformed themselves (Bottcher et al., 2018) at gene and transcriptome levels (Bi et al., 2021; Sun et al., 2021) to shape a surrounding which is suitable for proliferation and differentiation. In this part, we discussed how the changes of p53 influence the cytokines secreting, suppressive ligands expression, and immunocytes with inhibitive function differentiation (Figure 3).

### The p53 mutation or deletion induces immunosuppressive cytokines and downregulates proinflammatory factors

Cytokines are the main proteins produced and secreted by many different cell types. They mediate immune system responses (Borish and Steinke, 2003) and communication between cells and immune system components (Propper and Balkwill, 2022). For one thing, cytokines can act directly on tumor cells to promote or inhibit their growth; for another, they can also influence the status of tumors by recruiting immune cells or stromal cells. Responses caused by different cytokines also have synergy or confrontation effects (Salazar-Onfray et al., 2007). All above combined the complexity of cytokines therapy (Conlon et al., 2019). Understanding the regulatory mechanism of cytokines may help enhance clinical benefits and reduce adverse reactions. Using RNA interference to reactivate p53 briefly in the p53-dificient mouse liver carcinoma model, (Xue et al., 2007) found that tumor proliferation is restricted and dependent on the cellular senescence program and consequently increased inflammation cytokines. We hypothesized that the influence of p53 in different status on tumor immunity may be achieved through the influence of cytokines.

Type I IFN (IFN I) is an important cytokine whose response plays significant roles in antiviral innate immune and anti-tumor adaptive immune (Woo et al., 2015). One of the mechanisms to active immunity is to upregulate the expression of IFN-stimulated genes (ISGs), thereby giving rise to ISG DC cells resembling type 1 DC cells. ISG DC cells present intact tumor-derived peptide-MHC I to reactivate antitumor immunity (Corrales et al., 2016; Duong et al., 2022). The attenuation of IFN I response is conducive to tumor evasion. Current studies confirmed that IFN I response is activated by stimulator of IFN genes (STING) pathway. Cells with mutp53 suppress downstream signaling of cGAS/STING. Mutp53 prevents STING-IRF3-TBK1 trimeric complex formation and IFN regulatory factor 3 (IRF3) activation *via* interacting with TANK binding protein kinase 1 (TBK1) (Balka et al., 2020; Ghosh et al., 2021), consequently downregulates the release of IFN I.

Interleukin-6 (IL-6) is an important trigger of tumor-promoting inflammation. Tumor cells exposed to IL-6 activate the oncogenic STAT3 transcription factor to promote epithelial-to-mesenchymal transition (EMT), which is the first step for tumor cell migration. MiR-34a, activated by p53, is a major inhibitor through targeting IL-6R (Rokavec et al., 2014). In studies investigating aging and carcinogenesis, it was found that pre-malignant epithelial cells induce EMT through a paracrine mechanism of IL-6 and IL-8. IL-6, and IL-8 caused the loss of p53 in normal cells and the p53 deficiency exacerbated the pro-malignant secretory activity, forming a vicious cycle (Downward et al., 2008). Whereas co-expression of wt-p53 and NF-κB in tumor associated macrophages (TAMs) can enhance the survival of tumor cells through secreting IL-6, CXCL-1, and promoting tumor associated neutrophils recruitment (Lowe et al., 2014).

Transforming growth factor-β (TGF-β) is an immunosuppressive cytokine that has both positive and negative roles in tumor formation (Yang et al., 2010). The canonical response of TGF-β is the phosphorylation of SMAD2 and SMAD3, which then combine with SMAD4 to mediate growth inhibition (Yang et al., 2010). TGF-β acts as a tumor suppressor and the loss of TGF-β signaling effectors is the molecular basis to develop tumors. A study in prostate cancer discovered the TGF-βRII and Smad4 in tumor cells taper off during the progression process (Zeng et al., 2004). TGF-β also acts as a tumor promoter owing to its immune suppressive effects (Batlle and Massague, 2019). TGF-β inhibits the differentiation and proliferation of effector T cells (Teffs) but enhances the fraction of regulatory T cells (Tregs) and other suppressive cells through the phosphorylation of Smad family proteins. Blocking TGF-β in breast cancer cell lines was effectively to counteract its effects (Yi et al., 2021).

The dual functions of TGF-β in tumor cell survival are interconnected with different status of p53. TGF-β mediates the inhibition of p53 and DNA damage response to conduce to tumor progression. These effects are achieved through the downstream signals of miR-100 and miR-125b upregulated by SMAD2/3 transcription factors (Ottaviani et al., 2018). Furthermore, TGF-β1 antagonizes p53-induced apoptosis in precancerous cells *via* switching the viral E2-associated factor 4 (E2F-4)/p107 complex to Smad/E2F-4 corepressor, which represses transcription and translation of p53 (Lopez-Diaz et al., 2013). Equally, both wt-p53 and mutp53 regulate the TGF-β-mediated human lung and breast EMT by affecting TGF-β/SMAD3-mediated signaling. By restricting the expression of Nox4, a NADPH oxidase, wt-p53 downregulates downstream focal adhesion kinase phosphorylation to reduce migration. On the contrary, mutp53 has a synergistic effect with TGF-β to upregulate Nox4 and promotes tumor cells evasion (Boudreau et al., 2014).

### The p53 mutation or deletion upregulates immunosuppressive ligands

Immunosuppressive ligands, also known as immune checkpoints, such as programmed cell death protein 1 (PD-1) and cytotoxic T lymphocyte-associated antigen-4 (CTLA-4), have attracted much attention since their discoverers won the 2018 Nobel Prize in Physiology or Medicine. Immune Checkpoints are inhibitory pathways to maintain the persistence of immune response and the stability of the internal environment based on self-tolerance (Abril-Rodriguez and Ribas, 2017). Currently, there are several immune checkpoint inhibitors (ICIs) proved by FDA for clinical treatment. The representative examples are ipilimumab for metastatic melanoma (Langer et al., 2007; Kirkwood et al., 2008; Weber et al., 2008), nivolumab for non-small cell lung cancer (Topalian et al., 2012; Hellmann et al., 2019; Forde et al., 2022) and avelumab for urothelial carcinoma (Powles et al., 2020; Powles et al., 2021). However, immune therapy still has limits. A large fraction of patients has no response to immune therapy and many patients responding develop drug resistance after several treatment cycles (Chowdhury et al., 2018). Understanding the intrinsic mechanisms of immune checkpoints expression can therefore deepen our understanding of tumor immune escape and help develop new treatment options.

It is discovered that PD-L1, PD-L2, and CTLA-4 expression is significantly increased in patients with TP53 mutations in both clinical samples and mouse models of hematologic neoplasms (Pascual et al., 2019; Sallman et al., 2020). Similar findings have been discovered in solid tumors (Thiem et al., 2019; Sun et al., 2020). Furthermore, other co-inhibitory receptors, such as PD-1, T-cell immunoglobulin and mucin-domain containing-3 (TIM3), and lymphocyte-activation gene 3 (LAG3) are co-expressed on tumor-infiltrating lymphocyte cells (Williams et al., 2019). Based on these specific subtypes of T cells and tumor cells, TP53 mutations are one of the indicators in predicting efficacy in patients treated with ICIs (Chen et al., 2019; Sun et al., 2020).

PD-L1 expression is regulated by multiple pathways. The gain-of-function mutant p53 upregulated IL-17 signaling and induced the transformation of infiltrating T cells into exhausted CD8^+^ T cells to counteract the effects of PD-1 inhibitors (Wang J. et al., 2021). Besides, PD-L1 expression is upregulated by IFN-γ induced immune response, and both wt- and mut-p53 work in this process. It isn’t the activity, but the expression level of p53 or mutp53 impacts PD-L1 expression activated by IFN-γ (Thiem et al., 2019). The non-coding RNA miR-34, transcriptionally induced by p53, is also proved to negatively regulate PD-L1 (Cortez et al., 2016). In addition, p53 transcriptionally induces the expression of PTEN gene, an inhibitor of PI3K/Akt/mTOR pathway, which downregulates PD-L1 and maintains immune response (Hays and Bonavida, 2019). For the p53 deletion losing inhibition of PD-L1, it is conducive to tumor immune evasion.

### The p53 mutation or deletion promotes immunosuppressive cells differentiation

Tumor immune microenvironment (TIME) refers to immunological components with tumors (Fu et al., 2021). Various immune cells are the major participators of immune response. Notably, patients with TP53 mutations display non-T cell infiltrated phenotype. The numbers of cytotoxic T cells, helper T cells, as well as NK cells significantly reduced. Meanwhile, highly immunosuppressive Tregs (Sallman et al., 2020) and M2 macrophages (Wang et al., 2021) are expanded in cases with TP53 mutations.

Some indirect evidence suggests different status of p53 impact the intensity of immune response. The adaptive immune response is enhanced in the mouse model of colon cancer treated with HDM201, a selective MDM2 inhibitor. After the HDM201 treatment, the percentage of DC cells and CD8^+^ T cells increased in a p53-dependent manner (Wang et al., 2021). Moreover, p53 activation combined with immune checkpoint blockade therapy has been found to make breakthroughs in a variety of tumors. Studies in hepatocellular carcinoma (HCC) demonstrated the number of infiltrating CD8^+^ T cells and the fraction of activated CD8^+^ T cells is significantly increased after the combination therapy (Xiao et al., 2022).

Tregs are the major suppressive cells in controlling immune tolerance and homeostasis of immune system. Tregs are considered as tumor-promoting cells (Ye et al., 2012) because of suppressing anti-tumor Teffs response by releasing inhibitory cytokines such as IL-10, TGF-β, and IL-35 (Li et al., 2020). The relationship between Tregs and p53 is complex. Patients treated with p53 vaccination displayed decreased frequencies of Tregs and the 2-year disease-free survival reached 88% (Schuler et al., 2014). High doses of p53-derived peptide inhibited the Tregs differentiation and immunosuppressive function *in vitro* (Mandapathil et al., 2013). However, a research found the lack of p53 in rheumatoid arthritis compromised Tregs differentiation because of the decreasing activity of STAT-5 (Park et al., 2013). Depletion of highly activated and strongly suppressive tumor-infiltrating Tregs contributes to clinical outcomes of immunotherapy (Van Damme et al., 2021). These phenomena suggest that wt-p53 in the normal state promotes Tregs differentiation to control immune response, but under the tumor environment, p53 tends to suppress Tregs action to limit tumors.

The suppressive effects in Tregs cells is mainly dependent on the expression and function of the transcription factor forkhead box P3 (Foxp3) (Ohkura and Sakaguchi, 2020). Further, the lineage stability of Tregs is closely related to the PI3K/Akt pathway and its suppressor PTEN (Huynh et al., 2015). As is mentioned above, the lack of p53 consequently promotes the accumulation of inactivated PTEN, thus activating PI3K/Akt signaling and the Tregs differentiation (Kang et al., 2017). Foxp3 also can be regulated by miR-149-39. A study in esophageal cancer found long non-coding RNA (lncRNA) maternally expressed gene 3 (MEG3) upregulates MDM2, the inhibitor of p53. The decreased p53 is unable to generate sufficient miR-149-3p to limit transcription of Foxp3, but upregulate Tregs (Xu et al., 2021).

Myeloid-derived suppressor cell (MDSC) is another important member of the suppressive immune microenvironment, which produces reactive oxygen species and other cytokines to inhibit T cell mediated immune response (Li et al., 2021). p53 mediates the quantity and quality of MDSC in TIME. The p53 deletion enhanced the recruitment of suppressive myeloid CD11b^+^ cells through upregulating the expression of CXCR3/CCR2-associated chemokines and macrophage colony-stimulating factor (M-CSF) (Blagih et al., 2020). The destabilizing p53 prevents MDSCs from ferroptosis through upregulating Heme Oxygenase-1 (Hmox1) expression to suppress lipid reactive oxygen species production (Zhu et al., 2021). The dysfunctional p53 promotes the expansion of lymphoid-like stromal network, which increased the expression of CXCL1, CCL3, and CCL21 to recruit more immunosuppressive populations, especially MDSCs (Guo et al., 2013).

TAMs are major tumor-infiltrating cells mediated a variety of cellular activities such as tumor cytotoxicity (Chen et al., 2018), angiogenesis, and lymphangiogenesis (Volk-Draper et al., 2019). TAMs can be simply divided into tumor killing M1 type and tumor promoting M2 type based on their response to tumors. The transform mechanism between M1 and M2 that remains a mystery is a research hotspot, and p53 plays a role in the process. As mentioned above, the co-expression of p53 and NF-κB in TAMs promotes the secretion of IL-6 and CXCL-1 to promote tumor cell survival (Lowe et al., 2014). Tumor cells lack p53 also release WNT ligands to stimulate TAMs to produce IL-1β. The IL-1β triggers an inflammatory cascade throughout the body and drives tumor metastasis (Wellenstein et al., 2019). In macrophages, p53 acetylation induced M1 polarization to maintain iron homeostasis (Zhou et al., 2018). *In vitro* co-cultures of M0 macrophages with H358 (a p53-null cell line) exosomes demonstrated that exosome-induced M2 polarization may be p53 independent (Pritchard et al., 2020). Besides, upregulated wt-p53 altered miRNA levels in the exosomes and promoted macrophage repolarization towards a more pro-inflammatory/antitumor M1 phenotype (Trivedi et al., 2016). Equally, TAMs will affect p53 during the response. The expression of VEGF-C and its receptor VEGFR3 promoted by TAMs results in the loss of p53 and PTEN in tumor cells, which contributes to tumor resistance (Li et al., 2017).

## Target p53: New therapeutic strategies for immunotherapy

In recent years, immunotherapy including ICIs, cancer vaccination, and adoptive cell therapy has revolutionized the treatment of cancer. As introduced above, the p53 mutation or deletion play a central role in tumor immune evasion, so reactivating wt-p53 or restoring tumor suppressive function of mutp53 are promising anti-tumor immunotherapy strategies.

Scientists has appreciated the antigenic character of p53 since 1990s and developed p53-based vaccines (DeLeo and Appella, 2020). A novel phase Ib clinical trial of adjuvant p53 peptide-loaded DC cells demonstrated that the DC–p53 vaccine triggered a p53-specific immune response in 11 patients with head and neck squamous cell carcinoma, out of the 16 patients treated (Schuler et al., 2014). In another phase I trial, patients with platinum-resistant ovarian cancer received a combination regimen of a Modified Vaccinia Ankara vaccine delivering wild-type human p53 (p53MVA) and gemcitabine chemotherapy. It is found that p53MVA could elevate p53-reactive CD4 and CD8 T-cell responses, and patients with greatest expansion of T cells had longer progression-free survival (PFS) (Hardwick et al., 2018). Moreover, restoring p53 expression by p53 mRNA nanomedicine (Xiao et al., 2022) or directly introducing wt-p53 gene could reprogram the TME and sensitize tumors to anti-PD-1 therapy in mice experiments (Kim et al., 2019).

Targeting p53-MDM2 pathway is another strategy. Combination of reactivation of wt-p53 and ICIs is also proved to have a better anti-tumor effect. Wang and co-workers demonstrated that HDM201, a potent and selective second-generation MDM2 inhibitor, could trigger adaptive immunity and develop durable, antigen-specific memory T cells in a p53-dependent manner. Combination of HDM201 and PD-1/PD-L1 blockade is more efficient for complete tumor regressions (Wang et al., 2021). Similarly, Fang et al. (2019) also discovered that p53 activation by APG-115 would reduce the number and proportion of immunosuppressive M2 macrophage and has a synergistic effect with PD-1 blockade in anti-tumor.

It isn’t difficult to find from the previous studies that p53 has a strong connection with TIME (Hassin and Oren, 2022). The p53 mutation and deletion in tumor cells trends to form a tumor promoting microenvironment. Growing evidence indicated that p53 could be an effective target to deal with immune evasion.

## Concluding remarks

p53 is a star molecular which mediates multiple cellular activities of tumors and attracts much attention since its discovery. Scientists gradually realized the better treatment is to recover normal immune response, not only to enhance immune response. Owing to the essential role of p53 mutation and deletion in tumor immune evasion, reactivation of expression and function of p53 to reshape TIME and restore anti-tumor immunity may be efficient treatment for anti-tumor. Although scientists have spent almost 30 years to develop p53-based therapies, p53 is still a mystery protein attracting our attention. Great expectations of targeting p53 and unstable efficacy urge us to have a deeper understanding of it.

## Glossary

NK cell: Natural killer cell
DC cell: Dendritic cell
Mutp53: Mutant p53
Wt-p53: Wild-type p53
MHC: Major histocompatibility complex
TAP: Transporter associated with antigen processing
ERAP1: Endoplasmic reticulum aminopeptidase 1
Myc: Myelocytomatosis
TLR3: Toll-like receptor 3
ERV: Endogenous retroviruses
IFN: Interferon
STAT: Signal transducer and activator of transcription
TCR: T cell receptor
TNF: Tumor necrosis factor
AML: Acute myeloid leukemia
TRAIL-R1/2: Tumor necrosis factor related apoptosis-inducing ligand receptor 1 and receptor 2
ISGs: IFN-stimulated genes
STING: Stimulator of IFN genes
IRF3: IFN regulatory factor 3
TBK1: TANK binding protein kinase 1
IL-6: Interleukin-6
EMT: Epithelial-to-mesenchymal transition
TAMs: Tumor accosicated macrophages
TGF-β: Transforming growth factor-β
Teffs: Effector T cells
Tregs: Regulatory T cells
E2F-4: Viral E2-associated factor 4
PD-1: Programmed cell death protein 1
CTLA-4: Cytotoxic T lymphocyte-associated antigen-4
ICIs: Immune checkpoint inhibitors
TIM3: T-cell immunoglobulin and mucin-domain containing-3
LAG3: Lymphocyte-activation gene 3
TIME: Tumor immune microenvironment
Foxp3: Factor forkhead box P3
LncRNA: Long non-coding RNA
MEG3: Maternally expressed gene 3
MDSC: Myeloid-derived suppressor cell
M-CSF: Macrophage colony-stimulating factor
Hmox1: Heme oxygenase-1
p53MVA: Modified vaccinia ankara vaccine delivering wild-type human p53
PFS: Progression-free survival.
